# Mechanisms for lesion localization in neuromyelitis optica spectrum disorders

**DOI:** 10.1097/WCO.0000000000000551

**Published:** 2018-04-26

**Authors:** Monika Bradl, Markus Reindl, Hans Lassmann

**Affiliations:** a Department of Neuroimmunology, Center for Brain Research, Medical University of Vienna, Vienna; b Clinical Department of Neurology, Medical University of Innsbruck, Innsbruck, Austria

**Keywords:** aquaporin 4, autoantibodies, myelin oligodendrocyte glycoprotein, neuromyelitis optica spectrum disorders, T cells

## Abstract

**Purpose of review:**

Neuromyelitis optica spectrum disorders (NMOSD) are severe inflammatory diseases of the central nervous system (CNS), with the presence of aquaporin 4 (AQP4)-specific serum antibodies in the vast majority of patients, and with the presence of myelin oligodendrocyte glycoprotein (MOG)-specific antibodies in approximately 40% of all AQP4-antibody negative NMOSD patients. Despite differences in antigen recognition, the preferred sites of lesions are similar in both groups of patients: They localize to the spinal cord and to the anterior visual pathway including retina, optic nerves, chiasm, and optic tracts, and – to lesser extent – also to certain predilection sites in the brain.

**Recent findings:**

The involvement of T cells in the formation of NMOSD lesions has been challenged for quite some time. However, several recent findings demonstrate the key role of T cells for lesion formation and localization. Studies on the evolution of lesions in the spinal cord of NMOSD patients revealed a striking similarity of early NMOSD lesions with those observed in corresponding T-cell-induced animal models, both in lesion formation and in lesion localization. Studies on retinal abnormalities in NMOSD patients and corresponding animals revealed the importance of T cells for the very early stages of retinal lesions which eventually culminate in damage to Müller cells and to the retinal nerve fiber layer. Finally, a study on cerebrospinal fluid (CSF) barrier pathology demonstrated that NMOSD immunopathology extends beyond perivascular astrocytic foot processes to include the pia, the ependyma, and the choroid plexus, and that diffusion of antibodies from the CSF could further influence lesion formation in NMOSD patients.

**Summary:**

The pathological changes observed in AQP4-antibody positive and MOG-antibody positive NMOSD patients are strikingly similar to those found in corresponding animal models, and many mechanisms which determine lesion localization in experimental animals seem to closely reflect the human situation.

## INTRODUCTION

Neuromyelitis optica spectrum disorders (NMOSD) are severe inflammatory diseases of the central nervous system (CNS), with the presence of aquaporin 4 (AQP4)-specific serum antibodies (AQP4-abs) in the vast majority of patients [1,2], and with the presence of myelin oligodendrocyte glycoprotein (MOG)-specific antibodies (MOG-abs) in approximately 40% of all AQP4-abs^negative^ NMOSD patients [3,4]. These autoantibodies target AQP4 on astrocytes, choroid plexus epithelial cells, ependymal cells, and Müller cells, and MOG on the outermost surface of myelin sheaths (Table 1). 

In patients, AQP4-abs may persist for many years without causing clinical disease [5,6], although they readily detect AQP4 on the surface of transfected human embryonic kidney cells used as gold standard for NMOSD diagnosis [7]. Likewise, the presence of AQP4-abs [8,9] in the circulation of experimental rodents does not cause damage to CNS structures. The situation might be similar in patients with MOG-abs, as the long-term presence of MOG-abs in the circulation of transgenic mice is also benign and does not cause any clinically or pathologically detectable damage [10]. These observations already demonstrate the efficient separation of antibodies from their targets by the blood-brain/spinal cord barriers (BBB), which exclude about 99.9% of all antibodies from the CNS [11]. Hence, the presence of pathogenic AQP4-abs and MOG-abs in the serum is insufficient for the formation of NMOSD-typical lesions. Moreover, even when antibodies alone gain access to the CNS parenchyma, due to BBB dysfunction in young AQP4-abs^positive^ rats [8], due to intraparenchymal antibody injection in mice [12], or due to an opening of the BBB by insufficiently activated CNS antigen-specific T cells in AQP4-abs^positive^ rats [13], AQP4-expressing astrocytes remain intact. Hence, an open BBB and the presence of antibodies in the parenchyma are insufficient for the induction of the large tissue-destructive lesions seen in NMOSD patients. When the BBB is bypassed by intraparenchymal injection of both complement and antibodies, astrocytes are destroyed by complement-mediated cellular cytotoxicity (CDCC), and myeloid cells are activated and recruited to the site of tissue injury [12,14,15]. How is this barrier overcome *in vivo*? Based on research in experimental animals, we have a clear picture about the cells and additional humoral factors required for lesion formation.

First and foremost, CD4^+^ T cells are needed. These cells are found in early active NMOSD lesions defined by IgG and complement deposition on perivascular astrocytic endfeet in a rosette-like pattern, by ongoing AQP4 loss, or by neutrophil and macrophage recruitment and microglia activation [16]. Most importantly, some of these CD4^+^ T cells express the OX40 antigen as marker for recent activation [13]. T-cell activation within the CNS takes place in the perivascular space of blood vessels and is achieved via antigen presentation by major histocompatibility complex (MHC) class II^+^ perivascular macrophages/dendritic cells. When these so-called antigen presenting cells encounter sufficient amounts of a specific CNS protein or of its fragments, when they are able to process this protein to the small antigenic peptides recognized by specific CD4^+^ T cells in the context of MHC class II products, and when they are able to adequately present the MHC-peptide complexes to specific T cells, T-cell activation will ensue. The efficiency of this activation process strongly depends on the amount of antigen available, and on the ease of antigen processing and presentation [17,18]. The activated CD4^+^ T cells in the CNS parenchyma do not damage astrocytes [8,13,19,20] or myelin sheaths [21,22] but open the BBB for the large-scale entry of antibodies and complement. Then, AQP4-abs can bind to astrocytic endfeet at the perivascular glia limitans, fix complement, and initiate destruction of the astrocytes by CDCC [8,13,19,20]. Also MOG-abs will find ‘their’ antigen [21,22]. In some cases, this may cause only weak complement deposition on myelin sheaths, as seen in a recent human patient [23] and in experimental mice after intracerebral antibody and complement injection [24]. In other cases, this may cause robust complement deposition on and subsequent destruction of myelin sheaths, as seen with the pathogenic murine mAb 8–18C5 *in vivo*[21,22] and with one human patient-derived MOG-IgG preparation *in vitro*[25]. Both mechanisms might form the basis for the observation that the clinical outcome of MOG-abs-associated disease is often [26–31], but not always [32^▪▪^,^33^] better as compared with AQP4-abs-associated disease.

### Lesion formation is facilitated by the induction of a proinflammatory milieu

For example, the local production of interferon-gamma by CNS infiltrating T cells could lead to an upregulation of complement factors, a downregulation of complement inhibitors, and an upregulation of the Fc gamma receptor III essential for antibody-dependent cellular cytotoxicity (ADCC) by activated macrophages and microglia [13,34]. CNS infiltrating T cells may also trigger the recruitment and activation of macrophages and microglia [17], which produce IL-1 facilitating lesion formation [35], or produce inducible nitric oxide synthetase (iNOS) amplifying tissue damage [36]. The extent to which astrocytes contribute to the local proinflammatory milieu in NMOSD is still unclear. When astrocytes survive the binding of AQP4-abs for a longer period of time, as seen *in vitro* in the absence of ADCC or CDCC [37], and *in vivo* in some intracerebrally AQP4-abs-injected animals with slow progression of tissue destruction [12,38,39], they can produce IL-6 and other chemokines [40,41] which may open the BBB in a T-cell-independent way [15]. However, most lesions in NMOSD patients [42] and corresponding T-cell-based rat models [8,13,19,20] develop rapidly [42], and astrocytes might not have enough time to significantly contribute to the formation of a proinflammatory environment.

Early lesions in NMOSD patients [16] and rodent models [8,14] contain high numbers of neutrophils. These cells favor the interactions of CNS antigen-specific T cells with the BBB at the earliest time points of lesion formation [43–45] and are important amplifiers of lesion formation and growth [14,46].

All the evidence summarized above places activated CNS antigen-specific T cells at the center stage of lesion formation in NMOSD.

Based on experimental models of CNS inflammation, we know

- that the availability of an antigen for T-cell activation determines the site of lesion formation [47],

- that the activation of CNS antigen-specific T cells is necessary for lesion induction [13,17,18], and

- that the ratio between CNS antigen-specific T cells and pathogenic antibodies determines whether single large lesions resembling typical lesions in NMOSD or multiple sclerosis (MS), or multifocal small lesions resembling lesions in acute disseminated encephalomyelitis, form [21].

We also know that the sites of lesion formation are further affected by MHC and non-MHC genes, by sex, and by the mode of sensitization [48], and that the MHC haplotype may determine whether T cells recognize an antigen in its posttranslationally modified and/or unmodified form [49].

In the next part of this article, we will show how the knowledge obtained from experimental models translates to mechanisms of lesion localization in NMOSD.

**Box 1:**
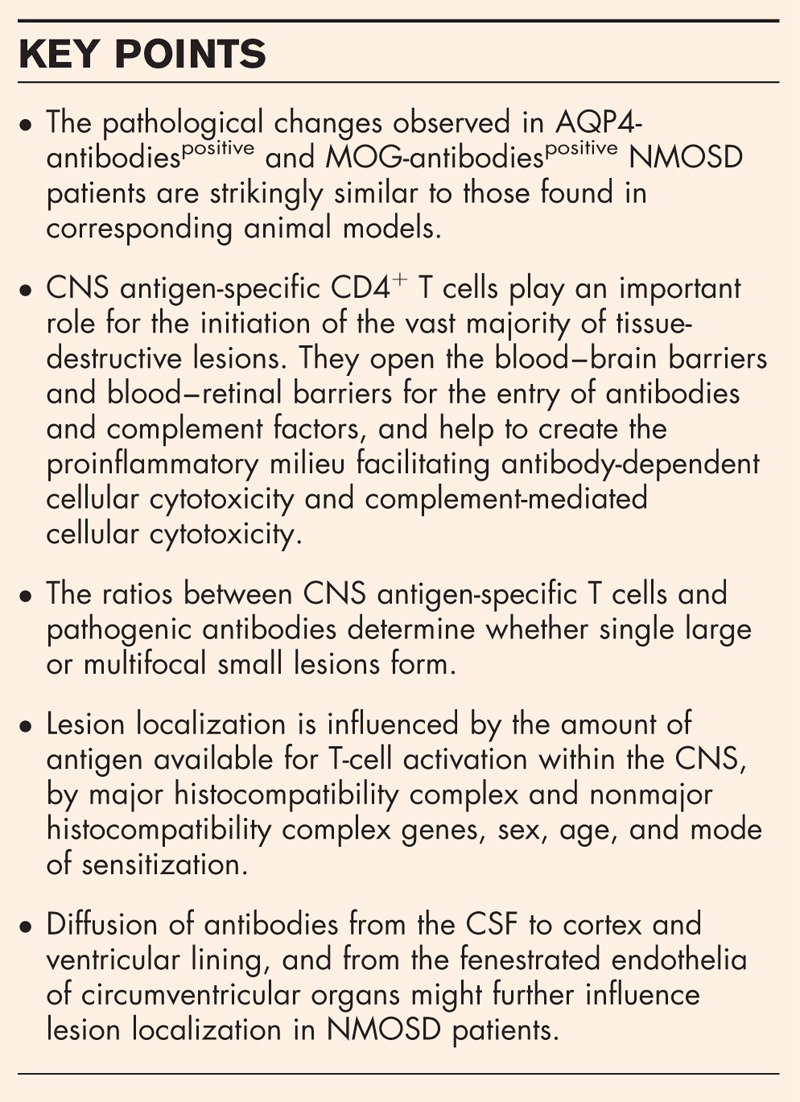
no caption available

## MECHANISMS DRIVING LESION LOCALIZATION IN SPINAL CORD AND OPTIC NERVES

The amounts of CNS antigens available for antigen presentation, T-cell activation, and antibody binding are the most important factors driving lesion formation in NMOSD, as spinal cord and optic nerves have higher AQP4 expression levels than the brain, both in humans and rats [50]. Moreover, the pathological changes in the spinal cord in the early course of NMOSD, that is the formation of perivascular lesions with AQP4 loss around radial vessels in the posterior and lateral columns, and the growth of lesions at the gray/white matter junction [51^▪▪^], are very similar to the changes observed in AQP4-abs^positive^ Lewis rats with T-cell-induced CNS inflammation [13,19,20,51^▪▪^,^52^,^53^].

In NMOSD patients, these lesions will then fuse with each other over time, extend towards the central spinal cord gray matter, and become larger in spinal cord gray matter, as this site contains higher numbers of AQP4-expressing astrocytes, translating to a higher availability of AQP4 for antigen presentation, T-cell activation, and antibody binding. Further lesion growth eventually culminates in spinal cord necrosis and atrophy [51^▪▪^].

The mechanisms underlying initiation and evolution of spinal cord lesions in NMOSD also recapitulate important aspects of the formation of brain lesions in MS [51^▪▪^]: In AQP4-ab^positive^ NMOSD, initial lesions preferentially form in the posterior and lateral columns which have a poor circulatory reserve, whereas in MS brains, plaques preferentially develop in hypo-perfused CNS white matter [51^▪▪^,^54^–^57^].

In AQP4-abs^positive^ NMOSD patients, astrocyte-destructive lesions with subsequent axonal damage also form in the anterior visual pathway containing the optic nerves, the chiasm, and the optic tracts, resulting from the extension of meningeal inflammatory infiltrates and AQP4 loss into the pial septa and parenchyma of these structures [58^▪▪^]. Lesions with AQP4 loss were also observed in the optic nerve and chiasm of AQP4-abs^positive^ T-cell-induced rat models of NMOSD [8].

Genetic differences between AQP4-abs^positive^ patient cohorts influence lesion location during onset attack. For example, longitudinally extensive spinal cord lesions were seen more frequently in UK whites, than in Afro-Caribbean or Japanese patients, whereas optic neuritis was more often found in Japanese patients than in UK whites or Afro-Caribbeans [59]. Genetic differences also translated to disease severity, with more severe onset attacks, a higher frequency of relapses, and a worse disease outcome in the UK cohort than in Japanese patients, and with more brain and multifocal lesions in Afro-Caribbean patients [59]. Also sex and age influence the disease outcome, with males and young onset patients being more likely to become visually disabled than females and old onset patients [59].

Optic neuritis is the most prominent disease manifestation in MOG-abs^positive^ patients, who often present with simultaneous optic neuritis and myelitis at onset [60], with optic neuritis at relapse [32^▪▪^], and with bilateral simultaneous optic neuritis [26]. In MRI, optic neuritis is associated with perineural soft tissue enhancement seen in up to 30% of cases [32^▪▪^,^61^–^63^]. MOG-abs^positive^ patients have spinal cord lesions in lower segments than AQP4-abs^positive^ patients, that is in the thoracic/lumbar cord and conus [26,64–66], and have either a normal brain MRI, or show nonspecific white matter lesions [26]. Lesions surrounding the third and fourth ventricle, subcortical lesions, and lesions in the cerebellar peduncle may occur, but are rare [26].

Again, there are striking similarities in plaque distribution between MOG-abs^positive^ patients and corresponding MOG-sensitized Brown Norway (BN) or Dark Agouti (DA) rats: Also in these animals, simultaneous lesions in both spinal cord and optic nerve, and bilateral loss of myelin from the optic nerves were seen, and there were cases with optic neuritis or with myelitis only [48]. Few animals developed destructive transverse myelitis or displayed additional small lesions in the cerebellar peduncles, the medulla oblongata, or other sites in the brain [48]. A very similar phenotype was observed in transgenic C57BL/6 mice expressing MOG-specific receptors on B and T cells [67,68]. In MOG-sensitized experimental animals, a profound influence of MHC and non-MHC genes on disease outcome and lesion localization was noted. For example, MHC genes determined the extent of demyelination [48,69], whereas non-MHC genes influenced the incidence of optic nerve involvement [48]. Genetic differences accounted for the higher incidence of simultaneously occurring lesions in spinal cord and optic nerves in BN vs. DA rats [48], and for the differences in response to the sensitization protocol [48]. Later work in congenic rat strains revealed that most of these differences were due to the influence of MHC class II genes [69,70]. Also sex differences became apparent: When DA rats were sensitized with the same protocol, 17/39 female rats and 0/11 male rats had lesions both in spinal cord and optic nerves [48].

To date, we have too little information about genetic differences in MOG-abs^positive^ patients to determine whether MHC or non-MHC genes influence lesion location. We only know that optic neuritis is the most prominent disease manifestation in several different ethnicities [26,27,32^▪▪^,^61^,^63^,^71^,^72^].

## MECHANISMS DRIVING LESION LOCALIZATION IN THE RETINA

For the longest time, retinal changes observed in AQP4-abs^positive^ NMOSD patients, that is the thinning of the retinal nerve fiber layer (RNFL) and the formation of cysts in the inner nuclear layer (INL), were considered a result of secondary retrograde degeneration after optic neuritis [73]. However, a recent study on human retinas indicated that the retina can also be a primary target in AQP4-abs^positive^ NMOSD [58^▪▪^]. This study revealed thinning of the RNFL and neuronal loss in the retinal ganglion cell layer of patients with a history of preceding optic neuritis, and ascribed these pathological changes to secondary retinal degeneration after optic neuritis [58^▪▪^]. However, they also identified retinal Müller cells as novel primary targets [58^▪▪^]. In three NMOSD retinas, Müller cells displayed loss of AQP4 reactivity in the absence of complement deposition. In addition, Müller cells and horizontal cells in the INL were reduced in numbers [58^▪▪^] which might provide a pathological explanation for the presence of microcystic INL abnormalities seen by optical coherence tomography in NMOSD patients [74]. Despite loss of AQP4 reactivity from Müller cells, AQP4 reactivity of astrocytes in the RNFL remained intact. There was no evidence for inflammatory T cells or B cells in these retinas [58^▪▪^]. Subsequent studies in AQP4-abs^positive^ NMOSD patients demonstrated foveal thinning irrespective of optic neuritis [75^▪^], even in different ethnic groups [75^▪^,76^▪^,^77^]. Hence, the retina is a primary target in AQP4-abs^positive^ NMOSD.

As Lewis rat models closely mimic the earliest steps of lesion formation in the spinal cord [8,20,51^▪▪^,78^▪^], they were used to identify the earliest steps of lesion formation in the retina. These animals showed retinal pathology only at sites of T-cell infiltration, suggesting that T cells opened the blood–retinal barriers (BRB) for the entry of antibodies. Müller cells showed ongoing loss of AQP4 reactivity, which required the presence of AQP4-abs, but was unrelated to CDCC or ADCC, whereas astrocytes in the RNFL remained intact [78^▪^,^79^]. At these earliest time points of retinal damage, the infiltrating T cells also recruited macrophages and activated microglial cells which expressed iNOS and were found in close vicinity to dysfunctional/damaged axons in the RNFL, indicating that not only Müller cells, but also the RNFL can be primary targets in NMOSD.

A second experimental model, in which the BRB was by-passed either by intravitreal injection of AQP4-abs in living rats, or by the addition of AQP4-abs (without complement or leukocytes) to retinal explant cultures [80^▪^], most likely reflects more advanced stages of retinal damage: Also in this model, retinal astrocytes continued to express AQP4, whereas Müller cells completely lost AQP4 reactivity which culminated in a secondary loss of retinal ganglion cells and a thinning of the ganglion cell complex 30 days after intravitreal injection of AQP4 [80^▪^]. These changes occurred independently of T-cell infiltration.

Cumulatively, data from animals and patients suggest that the opening of the BRB by inflammatory T cells is the first, important step for the induction of retinal damage, whereas further tissue damage might then progress in a T-cell-independent way. How long T cells are needed, and whether primary T-cell-induced inflammation may sufficiently activate astrocytes in the RNFL to produce IL-6 and thus cause a secondary opening of the BRB for the entry of antibodies [40,41] remains to be seen.

Also MOG-abs^positive^ patients [81^▪▪^,^82^,^83^] show a thinning of the RNFL, and a reduction in volume of the ganglion cell and inner plexiform layers, without any significant differences in parameters to AQP4-abs^positive^ patients [81^▪▪^]. In most cases, retinal damage in MOG-abs^positive^ patients occurs in eyes with a previous history of optic neuritis [81^▪▪^,^82^] and could thus reflect secondary degeneration, especially as the retina does not contain MOG expressing cells. Remarkably, however, a reduction in RNFL thickness was also observed in two fellow eyes without clinically evident previous optic neuritis [81^▪▪^]. The mechanisms underlying this finding are currently unresolved.

## MECHANISMS CAUSING THE PRESENCE OF ANTIBODIES IN THE CEREBROSPINAL FLUID

AQP4-abs are detectable in the cerebrospinal fluid (CSF) of most AQP4-abs seropositive NMOSD patients, most often when the patients had an acute disease relapse within 30 days prior to lumbar puncture [84]. This suggests that the antibodies reached this compartment in the course of CNS inflammation, possibly due to an opening of leptomeningeal vessels or subarachnoid veins by inflammatory T cells [85]. However, AQP4-abs titers in the CSF were also often proportional to those found in the serum, at the ratio of 1 (CSF) to 500 (serum) [86] which implies passive entry of antibodies from the serum. In rare instances, AQP4-abs are also synthesized in the CSF by plasmablasts [53,84], but it remains unclear whether these cells can access this compartment on their own, or whether they just survive there after onset attack or disease relapse. Recently, the choroid plexus was suggested as possible port of entry for pathogenic antibodies to the CSF [87^▪▪^]. Choroid plexus epithelial cells form the blood–CSF barrier, express AQP4 molecules on their basolateral surface, and were identified as targets for AQP4-abs and complement molecules entering the choroid stroma from the fenestrated endothelium of the choroid plexus vasculature. In this study, profound reduction or even loss of AQP4 reactivity by choroid plexus epithelial cells was seen, but the choroid plexus did not show any signs of damage [87^▪▪^], and a final proof for antibody access from the choroid plexus to the CSF is still missing.

MOG-abs in the CSF are also found in MOG-abs seropositive patients. In many cases, these antibodies seem to reach the CSF from the periphery [88^▪▪^], as there is only little evidence for intrathecal immunoglobulin synthesis [89,90].

Independently of the origin of AQP4-abs or MOG-abs found in the CSF, these molecules could reach several different target sites in the brain and could then further modify lesion localization in NMOSD.

## MECHANISMS DRIVING THE FORMATION OF CORTICAL LESIONS

AQP4-abs from the CSF could bind to the pial glia limitans, where loss or decrease of AQP4 reactivity was observed in ∼90% of NMOSD cases studied [87^▪▪^]. In the cerebral cortex, it coincided with vacuolation of pial and subpial tissue, and with the formation of enlarged spaces between astrocytic processes [87^▪▪^]. Diffusion of AQP4-abs within the underlying cortex could then be the pathological driver of the loss of AQP4 reactivity in cortical layer I, and the associated neuronal loss in cortical layers II–IV observed in NMOSD patients with cognitive impairment [87^▪▪^,^91^].

Juxtacortical lesions may also occur in MOG-abs^positive^ NMOSD patients [27], in which – to our knowledge – subcortical demyelination has not been described yet. However, in MOG-sensitized rats and marmoset monkeys, MOG-abs are involved in cortical demyelination, depending on particular combinations of MHC class I and class II isotypes and alleles [92,93].

## MECHANISMS DRIVING LESION LOCALIZATION TO THE VENTRICULAR LINING AND MEDULLA

Both in AQP4-abs^positive^ and MOG-abs^positive^ NMOSD patients, periventricular lesions may be found [26,27], most commonly around the third and fourth ventricle and the aqueduct of Sylvius [26,60,94]. A similar distribution of lesions is also seen in several different AQP4-abs^positive^[8,19,20] or MOG-abs^positive^[48,95] rat models. In these animals, areas of astrocyte and myelin loss have a perivascular pattern typical for T-cell-induced lesions. This lesion pattern is also observed in AQP4-abs^positive^ NMOSD patients [16]. An additional entry site of AQP4-abs was suggested by a recent study which demonstrated that AQP4-abs from the CSF may target the ependymal lining of ventricles. The authors observed morphological changes of ependymocytes and even loss of AQP4 reactivity from these cells, associated with subependymal gliosis and glial nodule formation [87^▪▪^]. They also noted complement deposition on ependymal cells in 38% of their NMOSD cases [87^▪▪^]. It remains unclear, whether these complement molecules derive from the CSF which they might have accessed in the course of CNS inflammation, whether they derive from perivascular inflammatory lesions formed close by, or whether they leaked from vessels with decreased barrier function in response to astrocytic IL-6 production [40]. In any case, the resulting damage of ependymal cells could open the gates for the entry of AQP4-abs from the CSF, causing or facilitating the formation of periventricular lesions [87^▪▪^].

As ependymocytes do not express MOG, this additional mechanism of periventricular lesion formation will not act in MOG-abs^positive^ patients.

### The medulla, finally, represents a special case for lesion formation

Medullary lesions are frequently observed in AQP4-abs^positive^ NMOSD cases, where they provide the pathological substrates for clinical symptoms like intractable hiccups and vomiting/nausea observed in about 40% of NMOSD patients [96]. Pathological studies of the medulla in AQP4-abs^positive^ NMOSD cases [97] and corresponding experimental models [8,20] revealed the formation of typical vasculocentric lesions with rosette-like complement deposition at many different sites in the medulla, and also from vessels adjacent to the area postrema. Moreover, MRI studies also documented an extension of lesions from the upper spinal cord in some [98], but not all [99] NMOSD patients. Jointly, these observations suggest that the vast majority of antibodies use a BBB opened by inflammatory T cells as entry route. However, the NMOSD-typical pattern of perivascular complement deposition was also documented in the area postrema, which contains fenestrated endothelial cells [95]. Medullary lesions may also occur in MOG-abs^positive^ patients, where they were seen by some [98], and not by other studies [26,27].The observed lesions located to the dorsal medulla/area postrema, and were also associated with intractable nausea and vomiting [100^▪▪^]. It remains to be seen whether T cells are involved in these process as well, or whether the area postrema provides an additional entry site for antibodies into the medulla.

## MECHANISMS DRIVING OR PREVENTING LESION FORMATION OUTSIDE THE CENTRAL NERVOUS SYSTEM

AQP4 is also expressed on syncytiotrophoblasts of the placenta, and symptoms like preeclampsia, intrauterine growth restriction, and stillbirth indicative of placental dysfunction are observed with increased frequency in AQP4-abs^positive^ NMOSD (for review refer to [101]). In fact, up to 43% of pregnancies in these patients are ended by miscarriages [102]. The underlying mechanism was elucidated in experimental animals, which revealed that AQP4-abs bind to syncytiotrophoblasts and activate complement, eventually culminating in placentitis. Severely inflamed placentas become necrotic, causing stillbirths and miscarriages, whereas less inflamed placentas are compatible with fetal survival [103]. There are many additional AQP4-expressing cells throughout the body, for example, cells of the lacrimal glands, salivary gland duct cells, Claudius, Hensen, and inner sulcus cells of the ear, olfactory epithelial cells, parietal acid-secreting cells of the stomach, airway cells of the lung, collecting duct principal cells of the kidney, and fast twitch fibers of skeletal muscles [104], to which pathogenic AQP4-abs also bind [9]. And yet, lesion formation at these sites is exceedingly rare, and so far only documented histologically for muscles of a single AQP4-abs^positive^ patient with recurrent myalgias and hyperCKemia [105]. The most likely explanation for this lack of extra-CNS lesions in AQP4-abs^positive^ NMO patients is the coexpression of AQP4 with complement regulatory proteins in peripheral organs [37].

Also MOG is expressed outside the CNS, as intracytoplasmatic antigen in Schwann cells [106]. However, as pathogenic MOG-abs do not recognize their antigen inside living cells, MOG-abs^positive^ patients are spared from additional, organ-specific autoimmune diseases.

## CONCLUSION

The pathological changes observed in AQP4-abs^positive^ and MOG-abs^positive^ NMOSD patients are strikingly similar to those found in corresponding animal models, suggesting shared mechanisms for lesion formation and localization.

## Acknowledgements

None.

### Financial support and sponsorship

The research studies of M.B. are supported by the Austrian Science Fund (FWF, P28476-B30 and I3335-B27). The research studies of M.R. are supported by research grants W1206 ‘Signal processing in neurons’ from the Austrian Science Fund (FWF), Bridge I project EDNA (FFG and Euroimmun), and research grants from the Austrian Multiple Sclerosis Research Society. H.L. and M.R. were also supported by the research grant ‘BIG-WIG MS’ from the Austrian Federal Ministry of Science, Research and Economy. The Neurological Research Laboratory (M.R., Medical University of Innsbruck and Tirol Kliniken) receives payments for antibody assays (AQP4 and antineuronal antibodies) and for MOG and AQP4 antibody validation experiments organized by Euroimmun (Germany).

### Conflicts of interest

There are no conflicts of interest.

## REFERENCES AND RECOMMENDED READING

Papers of particular interest, published within the annual period of review, have been highlighted as:

▪ of special interest
▪▪ of outstanding interest

## Figures and Tables

**Table 1:** The essentials of antigen recognition by pathogenic aquaporin 4-antibodies and pathogenic myelin oligodendrocyte glycoprotein-antibodies

The antibodies target antigens on the surface of cells (AQP4-abs) or of myelin sheaths (MOG-abs)
AQP4-abs recognize conformational epitopes which are formed by three extracellular loops of AQP4 and are further modified by the formation of AQP4 tetramers and orthogonal arrays of particles [107]
MOG-abs recognize conformational epitopes located at extracellular loops connecting the β strands of MOG [108]

abs, antibodies; AQP4, aquaporin 4; MOG, myelin oligodendrocyte glycoprotein.

## References

[1] Lennon VA Wingerchuk DM Kryzer TJ A serum autoantibody marker of neuromyelitis optica: distinction from multiple sclerosis. Lancet 2004; 364:2106–2112. 15589308 10.1016/S0140-6736(04)17551-X

[2] Lennon VA Kryzer TJ Pittock SJ IgG marker of optic-spinal multiple sclerosis binds to the aquaporin-4 water channel. J Exp Med 2005; 202:473–477. 16087714 10.1084/jem.20050304 PMC2212860

[3] Hamid SHM Whittam D Mutch K What proportion of AQP4-IgG-negative NMO spectrum disorder patients are MOG-IgG positive? A cross sectional study of 132 patients. J Neurol 2017; 264:2088–2094. 28840314 10.1007/s00415-017-8596-7 PMC5617862

[4] Sepulveda M Aldea M Escudero D Epidemiology of NMOSD in Catalonia: influence of the new 2015 criteria in incidence and prevalence estimates. Mult Scler 2017; DOI: 10.1177/1352458517735191. [Epub ahead of print]. 10.1177/1352458517735191 28984163

[5] Leite MI Coutinho E Lana-Peixoto M Myasthenia gravis and neuromyelitis optica spectrum disorder: a multicenter study of 16 patients. Neurology 2012; 78:1601–1607. 22551731 10.1212/WNL.0b013e31825644ff PMC3348852

[6] Nishiyama S Ito T Misu T A case of NMO seropositive for aquaporin-4 antibody more than 10 years before onset. Neurology 2009; 72:1960–1961. 19487655 10.1212/WNL.0b013e3181a82621

[7] Waters P Reindl M Saiz A Multicentre comparison of a diagnostic assay: aquaporin-4 antibodies in neuromyelitis optica. J Neurol Neurosurg Psychiatry 2016; 87:1005–1015. 27113605 10.1136/jnnp-2015-312601 PMC5013123

[8] Bradl M Misu T Takahashi T Neuromyelitis optica: pathogenicity of patient immunoglobulin in vivo. Ann Neurol 2009; 66:630–643. 19937948 10.1002/ana.21837

[9] Ratelade J Bennett JL Verkman AS Intravenous neuromyelitis optica autoantibody in mice targets aquaporin-4 in peripheral organs and area postrema. PLoS One 2011; 6:e27412. 22076159 10.1371/journal.pone.0027412 PMC3208637

[10] Litzenburger T Fassler R Bauer J B lymphocytes producing demyelinating autoantibodies: development and function in gene-targeted transgenic mice. J Exp Med 1998; 188:169–180. 9653093 10.1084/jem.188.1.169 PMC2525547

[11] Yu YJ Watts RJ Developing therapeutic antibodies for neurodegenerative disease. Neurotherapeutics 2013; 10:459–472. 23549647 10.1007/s13311-013-0187-4 PMC3701773

[12] Saadoun S Waters P Bell BA Intra-cerebral injection of neuromyelitis optica immunoglobulin G and human complement produces neuromyelitis optica lesions in mice. Brain 2010; 133:349–361. 20047900 10.1093/brain/awp309 PMC2822632

[13] Pohl M Kawakami N Kitic M T cell-activation in neuromyelitis optica lesions plays a role in their formation. Acta Neuropathol Commun 2013; 1:85. 24367907 10.1186/2051-5960-1-85 PMC3879999

[14] Saadoun S Waters P MacDonald C Neutrophil protease inhibition reduces neuromyelitis optica-immunoglobulin G-induced damage in mouse brain. Ann Neurol 2012; 71:323–333. 22374891 10.1002/ana.22686 PMC3643520

[15] Saadoun S Waters P Macdonald C T cell deficiency does not reduce lesions in mice produced by intracerebral injection of NMO-IgG and complement. J Neuroimmunol 2011; 235:27–32. 21492943 10.1016/j.jneuroim.2011.03.007

[16] Lucchinetti CF Mandler RN McGavern D A role for humoral mechanisms in the pathogenesis of Devic's neuromyelitis optica. Brain 2002; 125:1450–1461. 12076996 10.1093/brain/awf151 PMC5444467

[17] Kawakami N Lassmann S Li Z The activation status of neuroantigen-specific T cells in the target organ determines the clinical outcome of autoimmune encephalomyelitis. J Exp Med 2004; 199:185–197. 14734524 10.1084/jem.20031064 PMC2211765

[18] Kawakami N Nagerl UV Odoardi F Live imaging of effector cell trafficking and autoantigen recognition within the unfolding autoimmune encephalomyelitis lesion. J Exp Med 2005; 201:1805–1814. 15939794 10.1084/jem.20050011 PMC2213265

[19] Pohl M Fischer MT Mader S Pathogenic T cell responses against aquaporin 4. Acta Neuropathol 2011; 122:21–34. 21468722 10.1007/s00401-011-0824-0 PMC3120973

[20] Zeka B Hastermann M Hochmeister S Highly encephalitogenic aquaporin 4-specific T cells and NMO-IgG jointly orchestrate lesion location and tissue damage in the CNS. Acta Neuropathol 2015; 130:783–798. 26530185 10.1007/s00401-015-1501-5 PMC4654751

[21] Lassmann H Brunner C Bradl M Linington C Experimental allergic encephalomyelitis: the balance between encephalitogenic T lymphocytes and demyelinating antibodies determines size and structure of demyelinated lesions. Acta Neuropathol 1988; 75:566–576. 3259787 10.1007/BF00686201

[22] Linington C Bradl M Lassmann H Augmentation of demyelination in rat acute allergic encephalomyelitis by circulating mouse monoclonal antibodies directed against a myelin/oligodendrocyte glycoprotein. Am J Pathol 1988; 130:443–454. 2450462 PMC1880661

[23] Spadaro M Gerdes LA Mayer MC Histopathology and clinical course of MOG-antibody-associated encephalomyelitis. Ann Clin Transl Neurol 2015; 2:295–301. 25815356 10.1002/acn3.164 PMC4369279

[24] Saadoun S Waters P Owens GP Neuromyelitis optica MOG-IgG causes reversible lesions in mouse brain. Acta Neuropathol Commun 2014; 2:35. 24685353 10.1186/2051-5960-2-35 PMC3977893

[25] Peschl P Schanda K Zeka B Human antibodies against the myelin oligodendrocyte glycoprotein can cause complement-dependent demyelination. J Neuroinflammation 2017; 14:208. 29070051 10.1186/s12974-017-0984-5 PMC5657084

[26] Sato DK Callegaro D Lana-Peixoto MA Distinction between MOG antibody-positive and AQP4 antibody-positive NMO spectrum disorders. Neurology 2014; 82:474–481. 24415568 10.1212/WNL.0000000000000101 PMC3937859

[27] Kitley J Waters P Woodhall M Neuromyelitis optica spectrum disorders with aquaporin-4 and myelin-oligodendrocyte glycoprotein antibodies: a comparative study. JAMA Neurol 2014; 71:276–283. 24425068 10.1001/jamaneurol.2013.5857

[28] Hoftberger R Sepulveda M Armangue T Antibodies to MOG and AQP4 in adults with neuromyelitis optica and suspected limited forms of the disease. Mult Scler 2015; 21:866–874. 25344373 10.1177/1352458514555785 PMC4824843

[29] Sepulveda M Armangue T Sola-Valls N Neuromyelitis optica spectrum disorders: comparison according to the phenotype and serostatus. Neurol Neuroimmunol Neuroinflamm 2016; 3:e225. 27144216 10.1212/NXI.0000000000000225 PMC4841645

[30] Hacohen Y Mankad K Chong WK Diagnostic algorithm for relapsing acquired demyelinating syndromes in children. Neurology 2017; 89:269–278. 28615429 10.1212/WNL.0000000000004117

[31] Hennes EM Baumann M Schanda K Prognostic relevance of MOG antibodies in children with an acquired demyelinating syndrome. Neurology 2017; 89:900–908. 28768844 10.1212/WNL.0000000000004312

[32▪▪] Jarius S Ruprecht K Kleiter I MOG-IgG in NMO and related disorders: a multicenter study of 50 patients. Part 2: Epidemiology, clinical presentation, radiological and laboratory features, treatment responses, and long-term outcome. J Neuroinflammation 2016; 13:280. 27793206 10.1186/s12974-016-0718-0 PMC5086042

[33] Sepulveda M Armangue T Martinez-Hernandez E Clinical spectrum associated with MOG autoimmunity in adults: significance of sharing rodent MOG epitopes. J Neurol 2016; 263:1349–1360. 27147513 10.1007/s00415-016-8147-7 PMC5831396

[34] Ratelade J Asavapanumas N Ritchie AM Involvement of antibody-dependent cell-mediated cytotoxicity in inflammatory demyelination in a mouse model of neuromyelitis optica. Acta Neuropathol 2013; 126:699–709. 23995423 10.1007/s00401-013-1172-z PMC3890328

[35] Kitic M Hochmeister S Wimmer I Intrastriatal injection of interleukin 1 beta triggers the formation of neuromyelitis optica-like lesions in NMO-IgG seropositive rats. Acta Neuropathol Comm 2013; 1:5. 10.1186/2051-5960-1-5 PMC3776214 24252536

[36] Aboul-Enein F Weiser P Hoftberger R Transient axonal injury in the absence of demyelination: a correlate of clinical disease in acute experimental autoimmune encephalomyelitis. Acta Neuropathol 2006; 111:539–547. 16718350 10.1007/s00401-006-0047-y

[37] Saadoun S Papadopoulos MC Role of membrane complement regulators in neuromyelitis optica. Mult Scler 2015; 21:1644–1654. 25698168 10.1177/1352458515571446

[38] Asavapanumas N Ratelade J Verkman AS Unique neuromyelitis optica pathology produced in naive rats by intracerebral administration of NMO-IgG. Acta Neuropathol 2014; 127:539–551. 24190619 10.1007/s00401-013-1204-8 PMC3954950

[39] Asavapanumas N Verkman AS Neuromyelitis optica pathology in rats following intraperitoneal injection of NMO-IgG and intracerebral needle injury. Acta Neuropathol Commun 2014; 2:48. 24758159 10.1186/2051-5960-2-48 PMC4234989

[40] Takeshita Y Obermeier B Cotleur AC Effects of neuromyelitis optica-IgG at the blood-brain barrier in vitro. Neurol Neuroimmunol Neuroinflamm 2017; 4:e311. 28018943 10.1212/NXI.0000000000000311 PMC5173350

[41] Howe CL Kaptzan T Magana SM Neuromyelitis optica IgG stimulates an immunological response in rat astrocyte cultures. Glia 2014; 62:692–708. 24492996 10.1002/glia.22635 PMC5392242

[42] Bonnan M Valentino R Debeugny S Short delay to initiate plasma exchange is the strongest predictor of outcome in severe attacks of NMO spectrum disorders. J Neurol Neurosurg Psychiatry 2017; DOI: 10.1136/jnnp-2017-316286. [Epub ahead of print]. 10.1136/jnnp-2017-316286 29030418

[43] Aube B Levesque SA Pare A Neutrophils mediate blood-spinal cord barrier disruption in demyelinating neuroinflammatory diseases. J Immunol 2014; 193:2438–2454. 25049355 10.4049/jimmunol.1400401

[44] Carlson T Kroenke M Rao P The Th17-ELR+ CXC chemokine pathway is essential for the development of central nervous system autoimmune disease. J Exp Med 2008; 205:811–823. 18347102 10.1084/jem.20072404 PMC2292221

[45] Sayed BA Christy AL Walker ME Brown MA Meningeal mast cells affect early T cell central nervous system infiltration and blood–brain barrier integrity through TNF: a role for neutrophil recruitment? J Immunol 2010; 184:6891–6900. 20488789 10.4049/jimmunol.1000126

[46] Jacob A Saadoun S Kitley J Detrimental role of granulocyte-colony stimulating factor in neuromyelitis optica: clinical case and histological evidence. Mult Scler 2012; 18:1801–1803. 22495946 10.1177/1352458512443994

[47] Berger T Weerth S Kojima K Experimental autoimmune encephalomyelitis: the antigen specificity of T lymphocytes determines the topography of lesions in the central and peripheral nervous system. Lab Invest 1997; 76:355–364. 9121118

[48] Storch MK Stefferl A Brehm U Autoimmunity to myelin oligodendrocyte glycoprotein in rats mimics the spectrum of multiple sclerosis pathology. Brain Pathol 1998; 8:681–694. 9804377 10.1111/j.1750-3639.1998.tb00194.x PMC8098227

[49] Warnecke A Musunuri S N’Diaye M Nitration of MOG diminishes its encephalitogenicity depending on MHC haplotype. J Neuroimmunol 2017; 303:1–12. 28011088 10.1016/j.jneuroim.2016.11.008

[50] Matiello M Schaefer-Klein J Sun D Weinshenker BG Aquaporin 4 expression and tissue susceptibility to neuromyelitis optica. JAMA Neurol 2013; 70:1118–1125. 23857303 10.1001/jamaneurol.2013.3124

[51▪▪] Hayashida S Masaki K Yonekawa T Early and extensive spinal white matter involvement in neuromyelitis optica. Brain Pathol 2017; 27:249–265. 27082714 10.1111/bpa.12386 PMC8029352

[52] Kurosawa K Misu T Takai Y Severely exacerbated neuromyelitis optica rat model with extensive astrocytopathy by high affinity antiaquaporin-4 monoclonal antibody. Acta Neuropathol Commun 2015; 3:82. 26637322 10.1186/s40478-015-0259-2 PMC4670539

[53] Bennett JL Lam C Kalluri SR Intrathecal pathogenic antiaquaporin-4 antibodies in early neuromyelitis optica. Ann Neurol 2009; 66:617–629. 19938104 10.1002/ana.21802 PMC3180961

[54] Holland CM Charil A Csapo I The relationship between normal cerebral perfusion patterns and white matter lesion distribution in 1,249 patients with multiple sclerosis. J Neuroimaging 2012; 22:129–136. 21447022 10.1111/j.1552-6569.2011.00585.x

[55] Mahad DH Trapp BD Lassmann H Pathological mechanisms in progressive multiple sclerosis. Lancet Neurol 2015; 14:183–193. 25772897 10.1016/S1474-4422(14)70256-X

[56] Trapp BD Stys PK Virtual hypoxia and chronic necrosis of demyelinated axons in multiple sclerosis. Lancet Neurol 2009; 8:280–291. 19233038 10.1016/S1474-4422(09)70043-2

[57] Haider L Zrzavy T Hametner S The topograpy of demyelination and neurodegeneration in the multiple sclerosis brain. Brain 2016; 139:807–815. 26912645 10.1093/brain/awv398 PMC4766379

[58▪▪] Hokari M Yokoseki A Arakawa M Clinicopathological features in anterior visual pathway in neuromyelitis optica. Ann Neurol 2016; 79:605–624. 26836302 10.1002/ana.24608

[59] Kitley J Leite MI Nakashima I Prognostic factors and disease course in aquaporin-4 antibody-positive patients with neuromyelitis optica spectrum disorder from the United Kingdom and Japan. Brain 2012; 135:1834–1849. 22577216 10.1093/brain/aws109

[60] Kitley J Woodhall M Waters P Myelin-oligodendrocyte glycoprotein antibodies in adults with a neuromyelitis optica phenotype. Neurology 2012; 79:1273–1277. 22914827 10.1212/WNL.0b013e31826aac4e

[61] Kim SM Woodhall MR Kim JS Antibodies to MOG in adults with inflammatory demyelinating disease of the CNS. Neurol Neuroimmunol Neuroinflamm 2015; 2:e163. 26516628 10.1212/NXI.0000000000000163 PMC4608758

[62] Kageyama T Takeoka K Hirose M Diagnostic value of extensive perineural enhancement in patients with anti-MOG antibody-associated optic neuritis. J Neurol Sci 2017; 381:443.

[63] Zhou L Huang Y Li H MOG-antibody associated demyelinating disease of the CNS: a clinical and pathological study in Chinese Han patients. J Neuroimmunol 2017; 305:19–28. 28284341 10.1016/j.jneuroim.2017.01.007

[64] Wang JJ Jaunmuktane Z Mummery C Inflammatory demyelination without astrocyte loss in MOG antibody-positive NMOSD. Neurology 2016; 87:229–231. 27306633 10.1212/WNL.0000000000002844 PMC4940064

[65] Di Pauli F Mader S Rostasy K Temporal dynamics of anti-MOG antibodies in CNS demyelinating diseases. Clin Immunol 2011; 138:247–254. 21169067 10.1016/j.clim.2010.11.013

[66] Kaneko K Sato DK Nakashima I Myelin injury without astrocytopathy in neuroinflammatory disorders with MOG antibodies. J Neurol Neurosurg Psychiatry 2016; 87:1257–1259. 26800711 10.1136/jnnp-2015-312676

[67] Krishnamoorthy G Lassmann H Wekerle H Holz A Spontaneous opticospinal encephalomyelitis in a double-transgenic mouse model of autoimmune T cell/B cell cooperation. J Clin Invest 2006; 116:2385–2392. 16955140 10.1172/JCI28330 PMC1555668

[68] Bettelli E Baeten D Jager A Myelin oligodendrocyte glycoprotein-specific T and B cells cooperate to induce a Devic-like disease in mice. J Clin Invest 2006; 116:2393–2402. 16955141 10.1172/JCI28334 PMC1555670

[69] Weissert R Wallstrom E Storch MK MHC haplotype-dependent regulation of MOG-induced EAE in rats. J Clin Invest 1998; 102:1265–1273. 9739061 10.1172/JCI3022 PMC509110

[70] Weissert R de Graaf KL Storch MK MHC class II-regulated central nervous system autoaggression and T cell responses in peripheral lymphoid tissues are dissociated in myelin oligodendrocyte glycoprotein-induced experimental autoimmune encephalomyelitis. J Immunol 2001; 166:7588–7599. 11390515 10.4049/jimmunol.166.12.7588

[71] Bouzar M Daoudi S Hattab S Neuromyelitis optica spectrum disorders with antibodies to myelin oligodendrocyte glycoprotein or aquaporin-4: clinical and paraclinical characteristics in Algerian patients. J Neurol Sci 2017; 381:240–244. 28991690 10.1016/j.jns.2017.08.3254

[72] Siritho S Sato DK Kaneko K The clinical spectrum associated with myelin oligodendrocyte glycoprotein antibodies (anti-MOG-Ab) in Thai patients. Mult Scler 2016; 22:964–968. 26498263 10.1177/1352458515614093

[73] Bennett JL de Seze J Lana-Peixoto M Neuromyelitis optica and multiple sclerosis: seeing differences through optical coherence tomography. Mult Scler 2015; 21:678–688. 25662342 10.1177/1352458514567216 PMC4425816

[74] Gelfand JM Cree BA Nolan R Microcystic inner nuclear layer abnormalities and neuromyelitis optica. JAMA Neurol 2013; 70:629–633. 23529376 10.1001/jamaneurol.2013.1832

[75▪] Jeong IH Kim HJ Kim NH Subclinical primary retinal pathology in neuromyelitis optica spectrum disorder. J Neurol 2016; 263:1343–1348. 27142716 10.1007/s00415-016-8138-8

[76▪] Oertel FC Kuchling J Zimmermann H Microstructural visual system changes in AQP4-antibody-seropositive NMOSD. Neurol Neuroimmunol Neuroinflamm 2017; 4:e334. 28255575 10.1212/NXI.0000000000000334 PMC5322864

[77] Yamamura T Nakashima I Foveal thinning in neuromyelitis optica: a sign of retinal astrocytopathy? Neurol Neuroimmunol Neuroinflamm 2017; 4:e347. 28439528 10.1212/NXI.0000000000000347 PMC5395067

[78▪] Zeka B Hastermann M Kaufmann N Aquaporin 4-specific T cells and NMO-IgG cause primary retinal damage in experimental NMO/SD. Acta Neuropathol Commun 2016; 4:82. 27503347 10.1186/s40478-016-0355-y PMC4977668

[79] Zeka B Lassmann H Bradl M Muller cells and retinal axons can be primary targets in experimental neuromyelitis optica spectrum disorder. Clin Exp Neuroimmunol 2017; 8:3–7. 28344667 10.1111/cen3.12345 PMC5347906

[80▪] Felix CM Levin MH Verkman AS Complement-independent retinal pathology produced by intravitreal injection of neuromyelitis optica immunoglobulin G. J Neuroinflammation 2016; 13:275. 27765056 10.1186/s12974-016-0746-9 PMC5072328

[81▪▪] Pache F Zimmermann H Mikolajczak J MOG-IgG in NMO and related disorders: a multicenter study of 50 patients. Part 4: Afferent visual system damage after optic neuritis in MOG-IgG-seropositive versus AQP4-IgG-seropositive patients. J Neuroinflammation 2016; 13:282. 27802824 10.1186/s12974-016-0720-6 PMC5088645

[82] Havla J Kumpfel T Schinner R Myelin-oligodendrocyte-glycoprotein (MOG) autoantibodies as potential markers of severe optic neuritis and subclinical retinal axonal degeneration. J Neurol 2017; 264:139–151. 27844165 10.1007/s00415-016-8333-7

[83] Akaishi T Sato DK Nakashima I MRI and retinal abnormalities in isolated optic neuritis with myelin oligodendrocyte glycoprotein and aquaporin-4 antibodies: a comparative study. J Neurol Neurosurg Psychiatry 2016; 87:446–448. 25749692 10.1136/jnnp-2014-310206

[84] Jarius S Franciotta D Paul F Cerebrospinal fluid antibodies to aquaporin-4 in neuromyelitis optica and related disorders: frequency, origin, and diagnostic relevance. J Neuroinflammation 2010; 7:52. 20825655 10.1186/1742-2094-7-52 PMC2945323

[85] Engelhardt B Vajkoczy P Weller RO The movers and shapers in immune privilege of the CNS. Nat Immunol 2017; 18:123–131. 28092374 10.1038/ni.3666

[86] Takahashi T Fujihara K Nakashima I Antiaquaporin-4 antibody is involved in the pathogenesis of NMO: a study on antibody titre. Brain 2007; 130:1235–1243. 17449477 10.1093/brain/awm062

[87▪▪] Guo Y Weigand SD Popescu BF Pathogenic implications of cerebrospinal fluid barrier pathology in neuromyelitis optica. Acta Neuropathol 2017; 133:597–612. 28184993 10.1007/s00401-017-1682-1 PMC5348570

[88▪▪] Jarius S Ruprecht K Kleiter I MOG-IgG in NMO and related disorders: a multicenter study of 50 patients. Part 1: Frequency, syndrome specificity, influence of disease activity, long-term course, association with AQP4-IgG, and origin. J Neuroinflammation 2016; 13:279. 27788675 10.1186/s12974-016-0717-1 PMC5084340

[89] Körtvélyessy MD Breu M Pawlitzki MD ADEM-like presentation, anti-MOG antibodies, and MS pathology: two case reports. Neurol Neuroimmunol Neuroinflamm 2017; 4:e335. 28331892 10.1212/NXI.0000000000000335 PMC5350621

[90] Yanagida A Iizuka T Nagai T MOG-IgG-positive multifocal myelitis with intrathecal IgG synthesis as spectrum associated with MOG autoimmunity: two case reports. J Neurol Sci 2017; 382:40–43. 29111015 10.1016/j.jns.2017.09.020

[91] Saji E Arakawa M Yanagawa K Cognitive impairment and cortical degeneration in neuromyelitis optica. Ann Neurol 2013; 73:65–76. 23378324 10.1002/ana.23721

[92] Storch MK Bauer J Linington C Cortical demyelination can be modeled in specific rat models of autoimmune encephalomyelitis and is major histocompatibility complex (MHC) haplotype-related. J Neuropathol Exp Neurol 2006; 65:1137–1142. 17146287 10.1097/01.jnen.0000248547.13176.9d

[93] Kap YS Bauer J Driel N B-cell depletion attenuates white and gray matter pathology in marmoset experimental autoimmune encephalomyelitis. J Neuropathol Exp Neurol 2011; 70:992–1005. 22002426 10.1097/NEN.0b013e318234d421

[94] Pittock SJ Lennon VA Krecke K Brain abnormalities in neuromyelitis optica. Arch Neurol 2006; 63:390–396. 16533966 10.1001/archneur.63.3.390

[95] Lassmann H Bradl M Multiple sclerosis: experimental models and reality. Acta Neuropathol 2017; 133:223–244. 27766432 10.1007/s00401-016-1631-4 PMC5250666

[96] Takahashi T Miyazawa I Misu T Intractable hiccup and nausea in neuromyelitis optica with antiaquaporin-4 antibody: a herald of acute exacerbations. J Neurol Neurosurg Psychiatry 2008; 79:1075–1078. 18420727 10.1136/jnnp.2008.145391

[97] Popescu BF Lennon VA Parisi JE Neuromyelitis optica unique area postrema lesions: nausea, vomiting, and pathogenic implications. Neurology 2011; 76:1229–1237. 21368286 10.1212/WNL.0b013e318214332c PMC3068006

[98] Roemer SF Parisi JE Lennon VA Pattern-specific loss of aquaporin-4 immunoreactivity distinguishes neuromyelitis optica from multiple sclerosis. Brain 2007; 130:1194–1205. 17282996 10.1093/brain/awl371

[99] Misu T Fujihara K Nakashima I Intractable hiccup and nausea with periaqueductal lesions in neuromyelitis optica. Neurology 2005; 65:1479–1482. 16275842 10.1212/01.wnl.0000183151.19351.82

[100▪▪] Jarius S Kleiter I Ruprecht K MOG-IgG in NMO and related disorders: a multicenter study of 50 patients. Part 3: Brainstem involvement – frequency, presentation and outcome. J Neuroinflammation 2016; 13:281. 27802825 10.1186/s12974-016-0719-z PMC5088671

[101] Shosha E Pittock SJ Flanagan E Weinshenker BG Neuromyelitis optica spectrum disorders and pregnancy: interactions and management. Mult Scler J 2017; 23:1808–1817. 10.1177/1352458517740215 29154728

[102] Nour MM Nakashima I Coutinho E Pregnancy outcomes in aquaporin-4-positive neuromyelitis optica spectrum disorder. Neurology 2016; 86:79–87. 26581304 10.1212/WNL.0000000000002208 PMC4731292

[103] Saadoun S Waters P Leite MI Neuromyelitis optica IgG causes placental inflammation and fetal death. J Immunol 2013; 191:2999–3005. 23935196 10.4049/jimmunol.1301483 PMC4161708

[104] Verkman AS Anderson MO Papadopoulos MC Aquaporins: important but elusive drug targets. Nat Rev Drug Discov 2014; 13:259–277. 24625825 10.1038/nrd4226 PMC4067137

[105] Guo Y Lennon VA Popescu BF Autoimmune aquaporin-4 myopathy in neuromyelitis optica spectrum. JAMA Neurol 2014; 71:1025–1029. 24911400 10.1001/jamaneurol.2014.775

[106] Pagany M Jagodic M Schubart A Myelin oligodendrocyte glycoprotein is expressed in the peripheral nervous system of rodents and primates. Neurosci Lett 2003; 350:165–168. 14550920 10.1016/s0304-3940(03)00899-1

[107] Iorio R Fryer JP Hinson SR Astrocytic autoantibody of neuromyelitis optica (NMO-IgG) binds to aquaporin-4 extracellular loops, monomers, tetramers, and high order arrays. J Autoimmun 2013; 40:21–27. 22906356 10.1016/j.jaut.2012.07.008 PMC3509259

[108] Mayer MC Breithaupt C Reindl M Distinction and temporal stability of conformational epitopes on myelin oligodendrocyte glycoprotein recognized by patients with different inflammatory central nervous system diseases. J Immunol 2013; 191:3594–3604. 24014878 10.4049/jimmunol.1301296

